# The role of left and right hemispheres in the comprehension of idiomatic language: an electrical neuroimaging study

**DOI:** 10.1186/1471-2202-10-116

**Published:** 2009-09-15

**Authors:** Alice M Proverbio, Nicola Crotti, Alberto Zani, Roberta Adorni

**Affiliations:** 1 Department of Psychology, University of Milano-Bicocca, Via dell'Innovazione 10, 20126, Milan, Italy; 2 Institute of Bioimaging and Molecular Physiology, CNR, Segrate-Milan, Italy

## Abstract

**Background:**

The specific role of the two cerebral hemispheres in processing idiomatic language is highly debated. While some studies show the involvement of the left inferior frontal gyrus (LIFG), other data support the crucial role of right-hemispheric regions, and particularly of the middle/superior temporal area. Time-course and neural bases of literal vs. idiomatic language processing were compared. Fifteen volunteers silently read 360 idiomatic and literal Italian sentences and decided whether they were semantically related or unrelated to a following target word, while their EEGs were recorded from 128 electrodes. Word length, abstractness and frequency of use, sentence comprehensibility, familiarity and cloze probability were matched across classes.

**Results:**

Participants responded more quickly to literal than to idiomatic sentences, probably indicating a difference in task difficulty. Occipito/temporal N2 component had a greater amplitude in response to idioms between 250-300 ms. Related swLORETA source reconstruction revealed a difference in the activation of the left fusiform gyrus (FG, BA19) and medial frontal gyri for the contrast idiomatic-minus-literal. Centroparietal N400 was much larger to idiomatic than to literal phrases (360-550 ms). The intra-cortical generators of this effect included the left and right FG, the left cingulate gyrus, the right limbic area, the right MTG (BA21) and the left middle frontal gyrus (BA46). Finally, an anterior late positivity (600-800 ms) was larger to idiomatic than literal phrases. ERPs also showed a larger right centro-parietal N400 to associated than non-associated targets (not differing as a function of sentence type), and a greater right frontal P600 to idiomatic than literal associated targets.

**Conclusion:**

The data indicate bilateral involvement of both hemispheres in idiom comprehension, including the right MTG after 350 ms and the right medial frontal gyrus in the time windows 270-300 and 500-780 ms. In addition, the activation of left and right limbic regions (400-450 ms) suggests that they have a role in the emotional connotation of colourful idiomatic language. The data support the view that there is direct access to the idiomatic meaning of figurative language, not dependent on the suppression of its literal meaning, for which the LIFG was previously thought to be responsible.

## Background

Idiomatic language comprises 'traditional phrasings' that have a fixed form and convey a metaphorical and figurative meaning that goes beyond the strict literal sense of the words. Indeed, the overall meaning can hardly be derived from analysis of the constituent words and their semantic and syntactic properties [1]. The meaning of a figurative sentence such as "I have been treated with gloves" does not derive from a literal word-by-word analysis but from a higher-order lexical segmentation ("treated with gloves" = very kindly).

Overall, all figurative expressions share the property of conveying meaning that goes beyond the literal interpretation. They often employ similes, metaphors, personifications, hyperboles, onomatopoeias and symbolism. Figurative language is commonly used with the intention of adding colour and interest, to awaken the imagination; it is more suggestive than literal; it uses exaggerations or alterations to make a particular point.

It has been proposed that this extra-linguistic, more pragmatic component of idiomatic language involves right hemispheric functions to a greater extent than the left. Furthermore, it has been observed that idiomatic expressions are more salient and arousing, and their comprehension gives a sort of emotional satisfaction resulting from the awareness of sharing a jargon with a restricted community of sophisticated and polished speakers.

The first indication that the right hemisphere might play a crucial role in the comprehension of metaphors came from neuropsychological observations of a specific impairment in matching a word with a metaphorical connotative pictorial representation in right- vs. left-damaged patients (e.g. [2-4]. However, as correctly pointed out by Coulson [5], the use of pictorial material might be more problematic for right-damaged patients, who might present visuo-spatial or even perceptual deficits. Indeed, Papagno and colleagues [6] provided direct evidence that visuo-spatial deficits in right-damaged patients may impair their comprehension of unambiguous idioms in a picture-matching task.

In addition, other neuropsychological cases rule against the hypothesis of a predominant right hemispheric role in the comprehension of idiomatic expressions [7]. In detail, Tomkins tried to assess how unilateral right hemisphere brain-damaged patients were impaired in processing metaphorical aspects of word meaning. Ambiguous adjectives that could convey either a metaphorical or a literal meaning were used as target words in auditory lexical decision tasks. Targets were preceded by primes that were valid (related to the target's metaphorical or literal meaning), neutral, or unrelated. Prime-target pairs were presented in two attention conditions, designed to favour either relatively automatic (short SOA) or relatively effortful (long SOA) mental processing. Right-damaged patients performed similarly to left-brain-damaged and normal control subjects in the short SOA condition, while both kinds of patient were disadvantaged by the longer SOA in processing idiomatic language. Again, Giora and colleagues [8] found that right-damaged patients performed even better the left-damaged ones in a test of comprehension of highly conventional metaphors, and that the performance was negatively correlated with the extent of a lesion in the left middle temporal gyrus. It has also been shown by rTMS studies that the left temporal lobe has a role in the comprehension of figurative language [9,10].

In an ERP study [11], participants were subjected to an S1-S2 paradigm in which S1 consisted of visually-presented metaphorical sentences followed by a word that could or could not be defined by S1. The data showed that metaphorically-related S2 words elicited significantly higher N400 amplitudes than non-related S2 words. LORETA showed differential activity between the two S2 conditions in the right middle/superior temporal areas. One problem with this study is that the processing of metaphorical expressions was not compared with that of literal ones, so it remains unknown whether the same pattern of results would have been obtained with non-figurative language (a right middle temporal generator for N400). In this regard, it is interesting that Nobre and colleagues [12,13] have found a bilateral anterior medial temporal lobe generator for N400 to literal language, so metaphorical phrasing might possibly have induced a right-sided asymmetry in MTL activation.

A recent ERP study seems to suggest that the left and right hemispheres have similar sensitivities to metaphorical language [14]. The authors found no visual field differences in the N400 to low cloze probability for literal vs. idiomatic language and concluded that both hemispheres were sensitive to idiomatic aspects of language.

Again, an fMRI study [15] indicated bilateral hemispheric involvement in idiom comprehension, namely the engagement of a left and right sided neural network of structures including the bilateral inferior frontal gyri and the left middle temporal gyrus, while the right middle temporal gyrus was exclusively involved in the processing of metaphorical language. Using event-related functional magnetic resonance imaging (ER-fMRI), Stringaris and coworkers [16] demonstrated that literal and (rather conventional) English metaphorical sentences (of the "A is a B" type), which took equally long to read outside the scanner, recruited the LH when the task involved judgments of meaningfulness. However, they also showed that deriving meaning from metaphorical as opposed to literal sentences activated the left IFG (which was also recruited for non-meaningful sentences) and the left thalamus. A similar role of the lIFG for processing metaphorical sentences was indicated by the rTMS study of Pobric and coworkers [17], which showed how stimulation of the left inferior frontal gyrus disrupted processing of familiar metaphorical word pairs as well as literal ones.

The goal of our study was to investigate possible differences in the time course and neural bases of idiomatic vs. literal language processing, with particular focus on the specific role of the left and right hemispheres in the comprehension of idiomatic language.

Another major goal was to establish the timing of access to the metaphorical vs. literal meanings of sentences in order to elucidate whether metaphorical meanings are accessed only after the literal meaning has been rejected, as suggested for example by some rTMS and fMRI studies [10,18], or are directly accessed on the basis of previous context in parallel with the literal meaning (parallel hypothesis, described in [5,19]).

In the present study, ERPs were time-locked to the final words of idiomatic and literal sentences, as well as to the target words. Sentences were matched for length, number of words, familiarity, comprehensibility, imagery value and abstractness of final words. Sentences were followed by single words; in half the cases, these were semantically related to the previous context. The task consisted in deciding whether they were related or unrelated. The target words were matched for length, written frequency of use, and the abstractness/concreteness dimension.

For the way literal and idiomatic sentences were constructed (unpredictable endings plus idiomatic key located right at the sentence final part) we hypothesized that the onset of the earliest sentence-type effect might index the time point in which idiomatic meaning was accessed. An early latency effect (earlier than N400) would probably suggest that the idiomatic meaning of sentences is directly accessed and does not depend on the suppression of its literal meaning, therefore not requiring a left inferior frontal activation (know to be involved, besides syntactic processing, in the selection between alternative sentence meanings). We further hypothesized the N400 was affected by sentence type being larger to idiomatic sentences as predicted by previous literature. The unprecedented LORETA analysis applied to ERPs time-locked to sentence final words (not contaminated by decision making and motor processes, nor by pictorial visuospatial processes) was expected to get some light on the neural circuitry subserving the processing of idiomatic language, possibly including left and right hemispheric regions (particularly the right MTG described in several studies [15,18,20-23].

## Methods

### Subjects

Fifteen Italian University students (8 women and 7 men) participated in this experiment. Their mean age was 24 years. All had normal or corrected-to-normal vision and reported no history of neurological illness or drug abuse. Handedness was assessed by a laterality preference inventory [24] while eye dominance was determined by two independent practical tests. The data from two subjects were subsequently discarded before ERP averaging because of excessive eye movements, while the data from two others were discarded because of poor performance (more than 20% errors). The experiments were conducted with the understanding and written consent of each participant and in accordance with ethical standards (Helsinki, 1964). The experimental protocol was approved by the ethical and research review board of the National Research Council in Milan. Subjects earned academic credits for their participation.

### Stimuli

The stimuli consisted of 360 meaningful Italian sentences, half of which were followed by a semantically related word and the other half by an incongruous word (see Table 1 for some examples of sentences and target words). Half the sentences conveyed an idiomatic meaning and the other half a literal one. They were constructed so that the first part conveyed a neutral meaning (e.g. "I have been treated/cured") that did not predict the idiomatic/metaphorical nature of the whole, while the final part was either clearly literal ("with antibiotics") or metaphorical ("with gloves", which in Italian means "very kindly" and is the so-called idiomatic key). Since ERPs were time-locked to the sentence final words, this arrangement allowed us to monitor the time course of idiomatic/literal processing from the very onset.

**Table 1:** Examples of sentences as a function of associativeness and stimulus type.

**SENTENCE**	**IDIOMATIC KEY**	**TARGET WORD**
**IDIOMATIC**		
		**ASSOCIATED**
La morte di quel buon uomo mi	spezzò il cuore	tristezza
*The death of that good man*	*broke my heart*	*dolefulness*
		**NOT-ASSOCIATED**
Si è svegliato col	piede sbagliato	indagine
*He got up on the*	*wrong side of the bed*	*enquiry*

**LITERAL**		
		**ASSOCIATED**
Per risolvere il problema mi rivolsi a degli	esperti	consulenza
*To solve the problem I turned to some*	*experts*	*advice*
		**NOT- ASSOCIATED**
Non trovo più il mio	pennarello giallo	successo
*I cannot find my*	*yellow felt-tip pen*	*success*

The whole set of stimuli consisted of 360 sentences.

Idiomatic expressions included were as follows: (1) non-interpretable literally; for example expressions such as "to throw money out of the window", or "to be thick skinned" were not included; (2) both transparent (comprehended on the basis of their literal meaning, such as *in the twinkling of an eye*) and opaque (i.e., incomprehensible to naïve speakers, such as the Italian expression "mangiare la foglia" (to eat the leaf) meaning "to get wise"); (3) without a link to an underlying metaphor, for example expressions such as "mangiare come un porco" (to eat like a horse) or were not included; (4) moderate syntactic variability with the idiomatic key integrated in the sentence syntax - so proverbs or common ways of speaking such as "He who laughs last laughs best" were not included; (5) both decomposable and non-decomposable. Idiom decomposability distinguishes expressions in which the meaning is distributed over all the constituent words [5], for example "to talk until one is blue in the face", from fixed expressions that cannot be decomposed, e.g. "to give the cold shoulder". For expressions in which the meaning was distributed over all the constituent words (decomposable), the ERPs were time-locked to the idiomatic key (the point from which the figurative meaning arises), for example, FLY in "he would not hurt a fly". On the other hand, for the non-decomposable expressions, the ERPs were time-locked to the onset of the idiom, for example GOOD EYE in "Ask her for advice she has a good eye". Sentence compositionality was not experimentally manipulated in this study.

Sentences and target words were matched across categories in order to avoid differences in linguistic properties other than their idiomatic or literal nature. In detail, they were matched for overall number of words, number of final words; syntactic structure, written frequency of final words taken form the COLFIS corpus [25], length of final words (in # letters), abstractness/concreteness of final words, comprehensibility and familiarity of the overall sentence, length of target words, frequency of target words, and abstractness/concreteness of target words.

The COLFIS corpus comprises 3,798,275 words from contemporary written Italian texts, and represents those that are actually read rather than all possible written texts. It includes 1,836,119 entries taken from the most popular newspapers, 1,306,653 from periodicals and 655,503 from books.

For sentence final words, the two main types (idiomatic and literal) were matched, whereas the target words were matched across the four types (associated and non-associated idiomatic and literal sentences). Table 2 shows the data relative to the procedure of matching across sentence types (idiomatic vs. literal) for the final items and across the four word types (idiomatic associated or non-associated, literal associated or non-associated) for the target words. The abstractness variable was dichotomized by assigning 1 to concrete and 0 to abstract words.

**Table 2:** Matching of final items and target words for length, written frequency of occurrence and abstractness, according to sentence/word type and associativeness.

**Sentence/word type**	**Length** **(# letters)**	**Frequency of use** **(Colfis)**	**# Concrete items**
FINAL WORDS			

**Idiomatic**	10.4	248.7	125

**Literal**	11.2	258.5	120

TARGET WORDS			

**Idiom./A**	8.34	110.9	2

**Idiom./NA**	7.72	120.7	2

**Lit./A**	8.12	120.7	6

**Lit./NA**	7.91	114.9	0

Key: A = associated; NA = non-associated, Idiom. = idiomatic; Lit. = literal.

A one-way repeated measures ANOVA (2 levels: idiomatic, literal) on the frequencies of sentence final items (F1,178 = 0.51; p = 0.82) belonging to the two types of sentence showed no difference in frequency. Another one-way (4 levels: idiomatic associated or non-associated, literal associated or non-associated) repeated measures ANOVA (F3,267 = 0.95; p = 0.96) on the frequency scores of the four classes of target words showed no statistical difference.

The four sentence types were also matched for comprehensibility and familiarity by 12 judges of similar age and educational level to the experimental subjects. Each of the judges (6 men and 6 women) was given a booklet containing the set of 360 randomly ordered sentences. The participants were asked to indicate how often they had previously heard, read or used each expression, using a 5-point scale and circling the number and associated descriptive term (1 = never; 2 = rarely; 3 = sometimes; 4 = often; 5 = very often). For comprehensibility of the overall expression they were asked to judge how easily they fully comprehended the meaning of the sentence (1 = no comprehension; 2 = limited comprehension; 3 = fairly easy to comprehend; 4 = very easy to comprehend; 5 = extremely easy to comprehend).

The results showed that comprehension was quite good for all sentence types (4.8 points on average; min = 3.58; max = 5.00. The four sentence types did not differ statistically in comprehensibility as shown by a one-way repeated measures ANOVA (F3,267 = 1.96; p = 0.12) computed on comprehensibility ratings (Literal associated = 4.76, SD = 0.16; Literal non-associated = 4.77, SD = 0.15; Idiomatic associated = 4.81, SD = 0.21; Idiomatic non-associated = 4.81, SD = 0.16).

For familiarity of the expressions the ratings were also very similar and statistically identical across categories but lower in value (2.71 points on average; min = 1.25; max = 4.58), suggesting that non-trivial literal sentences should be selected to match perfectly with the idiomatic expressions. The familiarity ratings for the four sentence types were also subjected to a one-way (4 levels: idiomatic associated or non-associated, literal associated or non-associated) repeated measures ANOVA (F3,267 = 1.04; p = 0.4) and showed no statistical differences (Literal associated = 2.66, SD = 0.62; Literal non-associated = 2.68, SD = 0.63; Idiomatic associated = 2.72, SD = 0.63; Idiomatic non-associated = 2.80, SD = 0.58).

Idiomatic and literal expressions were also matched for cloze probability of the final parts. For this purpose, 15 judges (6 women and 9 men) of the same age and cultural level as the experimental subjects evaluated the cloze probability of each of the 360 sentences. They were presented with a booklet containing the whole set of sentences, randomly ordered, lacking the final part, and were asked to complete each with the first thing (1-3 words) that entered their minds. Analysis of the questionnaires showed no difference in the cloze probabilities of the two sentence types (idiomatic = 6.7%, SE = 12.7; literal = 8.1%, SE = 14.6). A repeated measures one-way ANOVA (2 levels: idiomatic, literal) confirmed the lack of statistical significance (F1,179 = 1.07; p = 0.317).

Overall, idiomatic expressions were not interpretable literally, were both opaque and transparent, were not metaphorical, were both decomposable and not decomposable, and their point recognition was located at the beginning of the sentence final word, exactly where the ERPs were time-locked.

Stimuli were randomly presented in the centre of a PC screen; longer sentences were presented in two centred lines. Sentences remained on the screen for 1 s and were then followed by a random ISI of 700-850 ms. This was followed by the final part of the sentence, which could make it either idiomatic or literal and was of 250 ms duration. After a random ISI of 900-1050 ms the target words followed, which could be either associated or not associated with the previous context and were of 250 ms duration. ITI was 1500 ms. Words were written in Arial Narrow font. The final words and the target words, on both of which the ERPs were time-locked, were in capital letters. All words were yellow on a grey background, 45' in height and from 2° 15' to 4° in length.

Participants sat comfortably in a darkened, acoustically and electrically shielded box in front of a computer screen located 72 cm from their eyes. They were instructed to fixate the centre of the screen and avoid any eye or body movements during the recording session.

The task consisted in deciding whether or not the target word was semantically associated with the previous sentence by pressing one button as accurately and rapidly as possible with the index finger or middle finger to signal a yes or no response (e.g., sentence: *Face the reality, do not play ostrich*, Target: *COWARDICE*. YES. Sentence: *The turtle has been cured and brought back to the water*: Target: *PROSE*. NO.). The two hands were used alternately during the recording session, and the hand and sequence order were counterbalanced across subjects.

### EEG recording and analysis

The EEG was continuously recorded from 128 scalp sites at a sampling rate of 512 Hz. Horizontal and vertical eye movements were also recorded. Linked ears served as the reference lead. The EEG and electro-oculogram (EOG) were amplified with a half-amplitude band pass of 0.016-100 Hz. Electrode impedance was kept below 5 kΩ. EEG epochs were synchronized with the onset of stimulus presentation and analyzed by ANT-*EEProbe *software. Computerized artefact rejection was performed before averaging to discard epochs in which eye movements, blinks, excessive muscle potentials or amplifier blocking occurred. EEG epochs associated with an incorrect behavioural response were also excluded. The artefact rejection criterion was peak-to-peak amplitude exceeding 50 μV, and the rejection rate was ~5%. ERPs were averaged off-line from -200 ms before to 1000 ms after stimulus onset. ERP components were identified and measured, with reference to the average baseline voltage over the interval from -100 ms to 0 ms, at sites and latency where they reached their maximum amplitude.

For ERPs elicited by final words, the mean area amplitude of the N2 component was measured at occipito/temporal sites (P9(T7), P10(T8), PPO9h (LO) and PP10h (RO) between 250 and 300 ms. The mean area amplitude of N400 was measured in the time window 360-550 ms at centro-parietal sites (CP3, CP4, P9, P10). The anterior P300 (or slow positivity) peak amplitudes and latencies were measured at frontal sites (F1, F2, F5, F6) between 500 and 780 ms.

For ERPs elicited by target words (associated or not associated), the mean area amplitude of centro-parietal N400 was measured at central (C1, C2) and lateral parietal (P7, P8) sites between 380 and 480 ms. The mean area of anterior N400 was measured at anterior frontal (AF7, AF8) and frontal (F1, F2) sites in the 410-510 ms time window. The slow positive component was quantified by mean amplitude measurements at central (C3, C4) and fronto-central (FCC5h, FCC6h) sites in the time window 500-650 ms.

Response times exceeding mean ± 2 standard deviations were excluded. Behavioural and ERP data were subjected to multifactorial repeated-measures ANOVA. The factors were "sentence type" (idiomatic, literal), "response hand" (left, right) and "semantic associativeness" (associated, not associated) for RT data, and additional "electrode", (dependent on ERP component of interest) and "hemisphere" (left, right) for ERP data. Multiple comparisons of means were done by post-hoc Tukey tests.

Topographical voltage maps of ERPs were produced by plotting colour-coded isopotentials obtained by interpolating voltage values between scalp electrodes at specific latencies. Low Resolution Electromagnetic Tomography (Pasqual-Marqui et al., 1994) was performed on grand-average ERP difference waves of interest at various time latencies using ASA3 and ASA4 software. LORETA, which is a discrete linear solution to the inverse EEG problem, corresponds to the 3D distribution of neuronal electric activity that has maximum similarity (i.e. maximum synchronization), in terms of orientation and strength, between neighbouring neuronal populations (represented by adjacent voxels). Source space properties were: grid spacing = 5-10 mm; estimated SNR = 3. In this study an improved version of the standardized sLORETA was used, which incorporates a singular value decomposition-based lead field weighting: swLORETA [26].

## Results

### Behavioural data

Responses to targets following literal sentences (538 ms) were faster than to idiomatic ones (560 ms) (F1,10 = 6.63, p < 0.028). They were also faster when the right hand (533 ms) rather than the less dominant hand (565 ms) was used, as shown by the statistical significance of "hand" (F1,10 = 21.7; p < 0.0009). Again, they were faster in response to associated (516 ms) than non-associated (582 ms) target words, as indicated by the "associativeness" factor (F1,10 = 11.91; p < 0.006).

The error percentage (mainly omissions) was quite low, but an ANOVA on the arcsin-transformed percentage of errors showed that "sentence type" was significant (F1,10 = 37.15; p < 0.00012), with a higher error percentage in response to targets following an idiomatic (8.2%) than a literal (4.8%) expression. Error percentages were also higher for associated (8.36%) than non-associated (4.68%) targets, as shown by the statistical significance of "associativeness" (F1,10 = 28.6; p < 0.0003).

### Electrophysiological data

#### ERPs to final words of idiomatic/literal expressions

**Figure 1:**
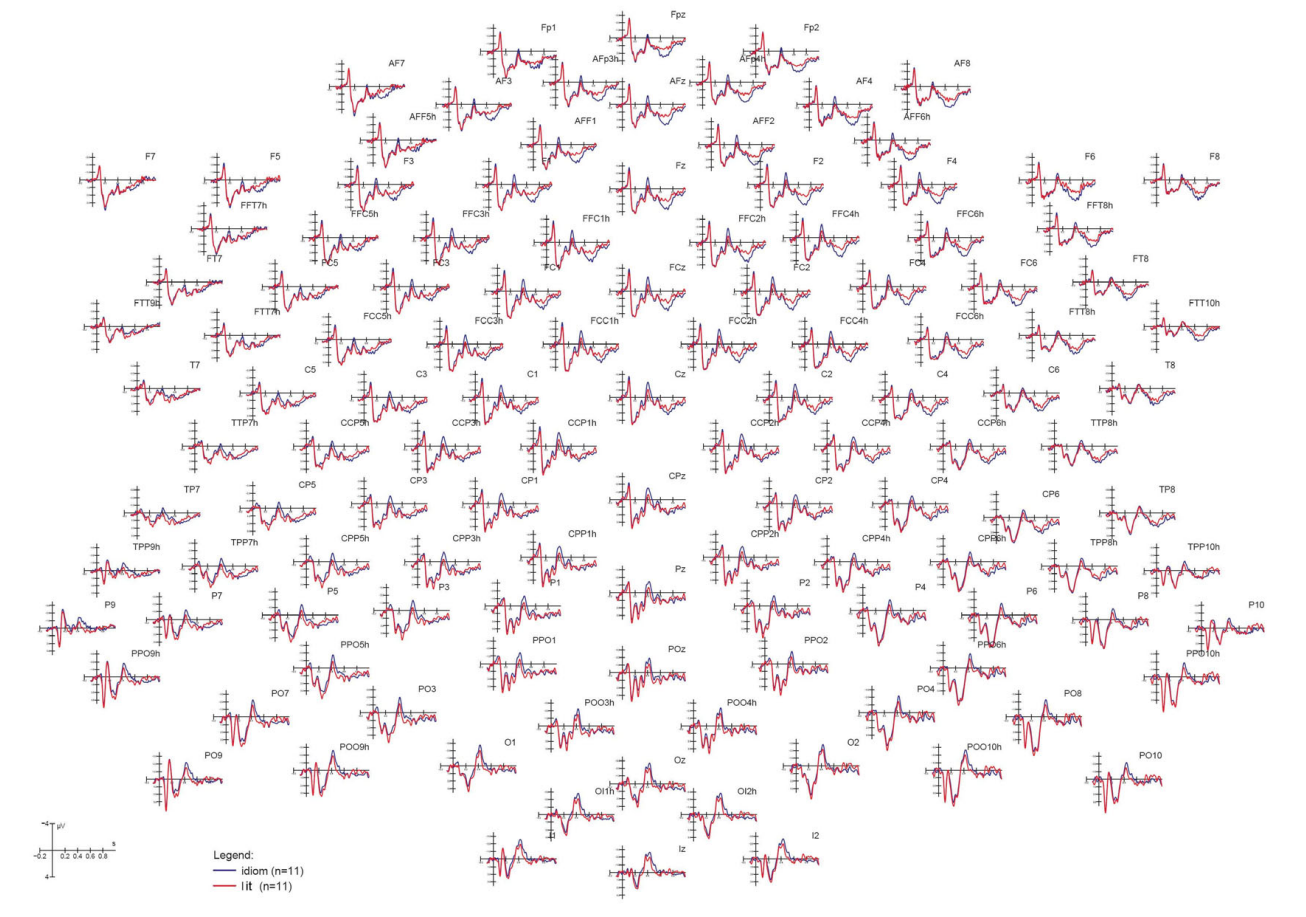
**Final words**. Grand-average ERPs recorded from all scalp sites as a function of sentence type.

Figure 1 shows the grand-average ERPs recorded from all scalp sites as a function of sentence type, irrespective of the associativeness of target words. Three main scalp areas affected by sentence type at various latency stages are visible: an occipito/temporal N2, a centro-parietal N400 and an anterior late positivity. ERP components of interest were selected accordingly.

#### N2 component

**Figure 2:**
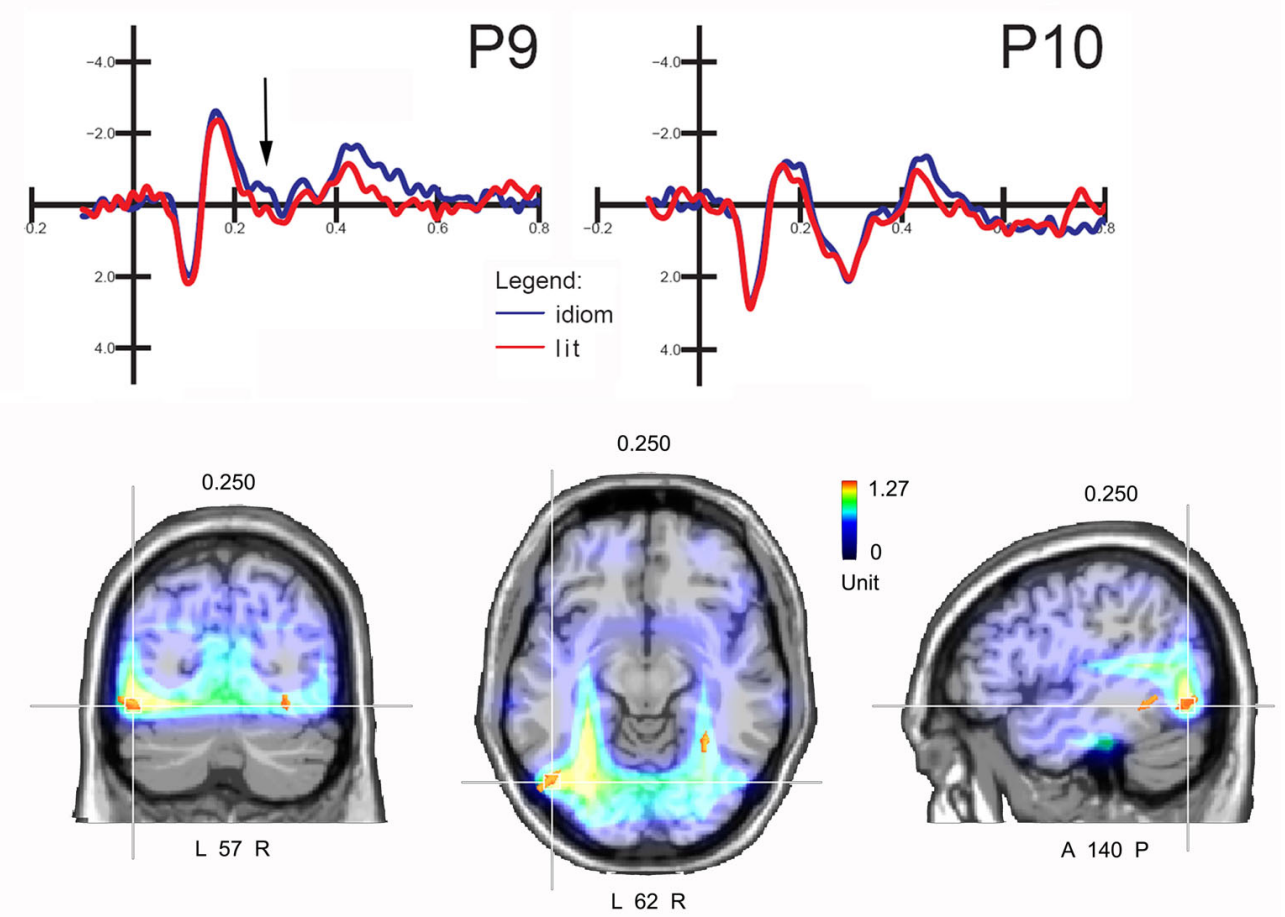
**Final words**. (Top) ERP recorded at left and right occipito/temporal sites as a function of sentence type. The arrows indicate the effect of sentence type on N2 amplitude over the left hemisphere. (Bottom) Coronal, axial and sagittal views of swLORETA source reconstruction computed on the difference wave idiomatic minus literal for the 270-300 ms time window. Grid spacing = 10 mm; estimated SNR = 3.

This component had greater amplitude over the left (2.45 μV) than the right (1.07 μV) hemisphere (F1,10 = 11.33; p < 0.008) and over the occipito/temporal area (F1,10 = 42.52; p < 0.00007). The interaction "sentence type" × "hemisphere" (F1,10 = 11.23; p < 0.007) indicated significantly greater N2 responses to idiomatic than to literal expressions over the left hemisphere. Figure 2 (top) shows grand-average ERPs recorded in response to idiomatic vs. literal expressions over the left and right posterior sites. A standardized weighted LORETA source reconstruction was performed on the difference wave obtained by subtracting ERPs to literal from ERPs to idiomatic expressions in the 270-300 ms time window (displayed in Figure 2, bottom). Table 3 shows a list of intracranial generators explaining the surface difference voltage, along with their Tailarach coordinates. The results indicated a strong focus of activation in the left fusiform gyrus (BA19, X = -48, Y = -66, Z = -11); significantly active sources were also found in the right fusiform gyrus (BA27) and in the left and right medial frontal gyri.

**Table 3:** Final words.

**Magn**.	**T-x**	**T-y**	**T-z**	**Hem**.	**lobe**	**area**	**BA**
10.27	-48	-66	-11	LH	Temporal	Fusiform gyrus	19

9.87	31	-46	-9	RH	Temporal	Fusiform gyrus	37

8.38	31	42	33	RH	Frontal	Medial frontal gyrus	9

5.88	-28	44	15	LH	frontal	Medial frontal gyrus	10

Tailarach coordinates corresponding to the intracranial generators explaining the difference voltages Idiomatic-Literal sentences in the 270-300 ms time window, according to swLORETA (ASA) [26]; grid spacing = 5 mm.

#### N400 component

### Latency

An ANOVA on N400 latency values showed no effect of sentence type or hemisphere on the latency of this negative deflection.

### Amplitude

**Figure 3:**
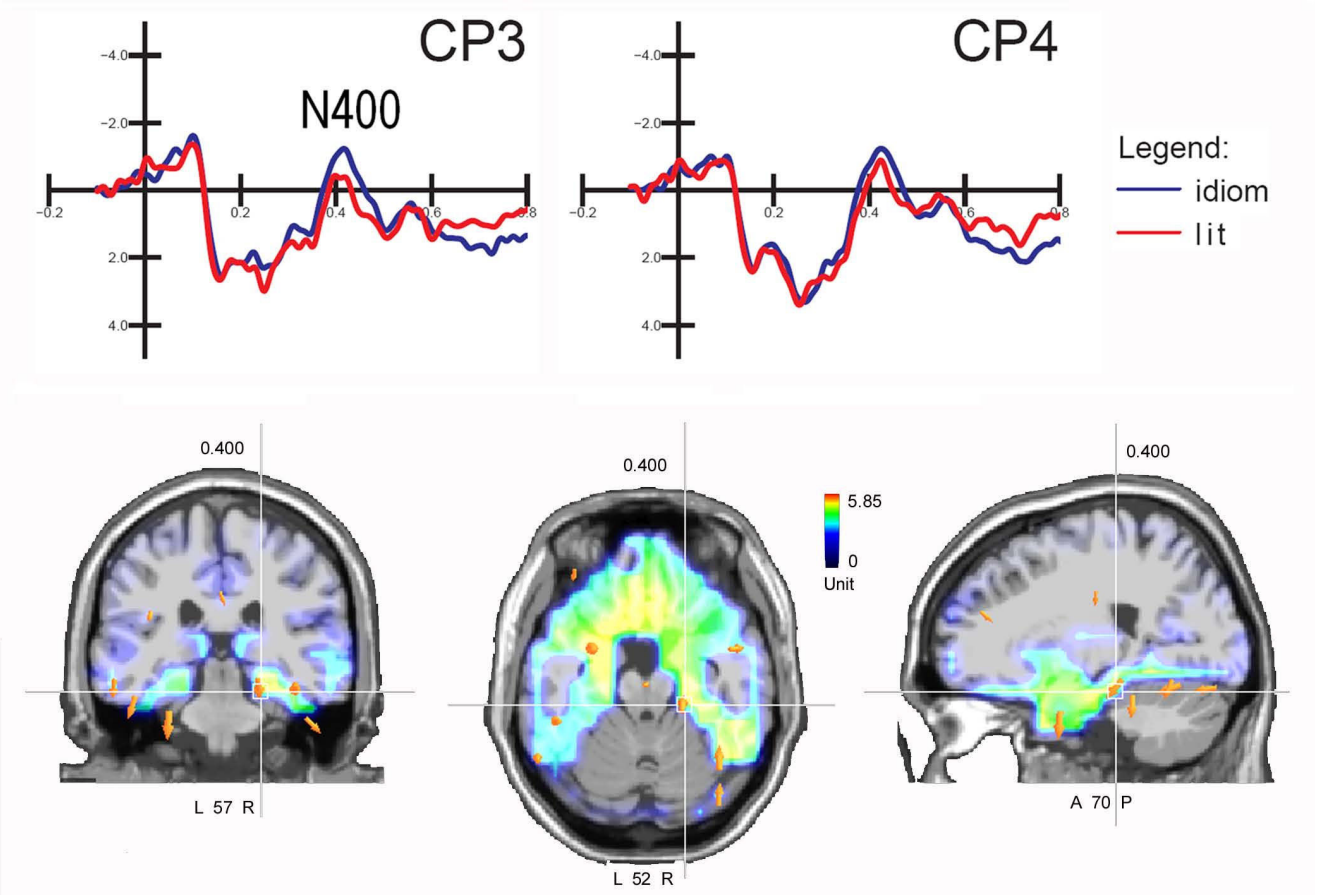
**Final words**. (Top) Grand-averaged ERPs recorded over centro-parietal sites in response to idiomatic and literal phrases. (Bottom) Coronal, axial and sagittal views of swLORETA inverse solution source performed on the difference wave obtained by subtracting ERPs to literal from ERPs to idiomatic sentences in the time window 400-450, corresponding to the peak of N400 response. Grid spacing = 5 mm; estimated SNR = 3.

N400 was strongly affected in amplitude by "sentence type" (F1,10 = 7.20; p < 0.023), being much larger to idiomatic (-2,19 μV) than to literal (-1,67 μV) expressions, as visible in the ERP waveforms of Figure 3 (top). A grand-average difference wave was computed by subtracting ERPs to literal from ERPs to idiomatic sentences. SwLORETA source reconstruction (see Figure 3B) was performed on the difference wave thus obtained in the time window 400-450 ms corresponding to the peak of the N400 response. Table 4 lists the intracranial generators that explain the difference voltage relative to the processing of idiomatic aspect of language. Significant active sources included, among others, the left and right fusiform gyri of the occipital lobe (BA20 and BA37), the left and right fusiform gyri of the temporal lobe (BA37 and B19), the left limbic regions (uncus and cingulate gyrus), the right parahippocampal region, the right middle temporal gyrus (X = 51, Y = -1, Z = -28, BA21) and the left middle frontal gyrus (X = -38, Y = 33, Z = 23, BA46).

**Table 4:** Final words.

**Magn**.	**T-x**	**T-y**	**T-z**	**Hem**.	**lobe**	**area**	**BA**
5.85	-28	-1	-28	LH	Limbic	Uncus	36

5.20	41	-55	-18	RH	Occipital	Fusiform gyrus	37

5.12	21	-24	-15	RH	Limbic	Parahippocampal gyrus	35

4.74	-48	-34	-24	LH	Occipital	Fusiform gyrus	20

4.68	41	-75	-19	RH	Temporal	Fusiform gyrus (11 mm)	19

4.23	51	-1	-28	RH	Temporal	Middle temporal gyrus	21

4.23	-58	-55	-18	LH	Temporal	Fusiform gyrus	37

31.14	-38	33	23	LH	Frontal	Middle frontal gyrus	46

3.00	1	-20	27	LH	Limbic	Cingulate gyrus	23

Tailarach coordinates corresponding to the intracranial generators explaining the difference voltages Idiomatic-Literal sentences in the 400-450 ms time window, according to swLORETA (ASA); grid spacing = 5 mm, magnitude in nA.

#### Anterior P3 (500-780 ms)

### Latency

The slow positive anterior component was earlier over the left hemisphere (F1,10 = 15.94; p < 0.002), especially at inferior frontal sites, as shown by the "electrode" × "hemisphere" interaction (F1,10 = 6.43; p < 0.03) and relative post-hoc comparisons (F5 = 597, F6 = 684 ms, p < 0.01; F1 = 653, F2 = 673 ms, n.s.). P3 latency was strongly affected by stimulus content, with earlier peaks to literal (640 ms) than to idiomatic (686 ms) phrases at the mesial frontal sites, as demonstrated by the significant "sentence type" × "electrode" interaction (F1,10 = 8.05; p < 0.02).

### Amplitude

**Figure 4:**
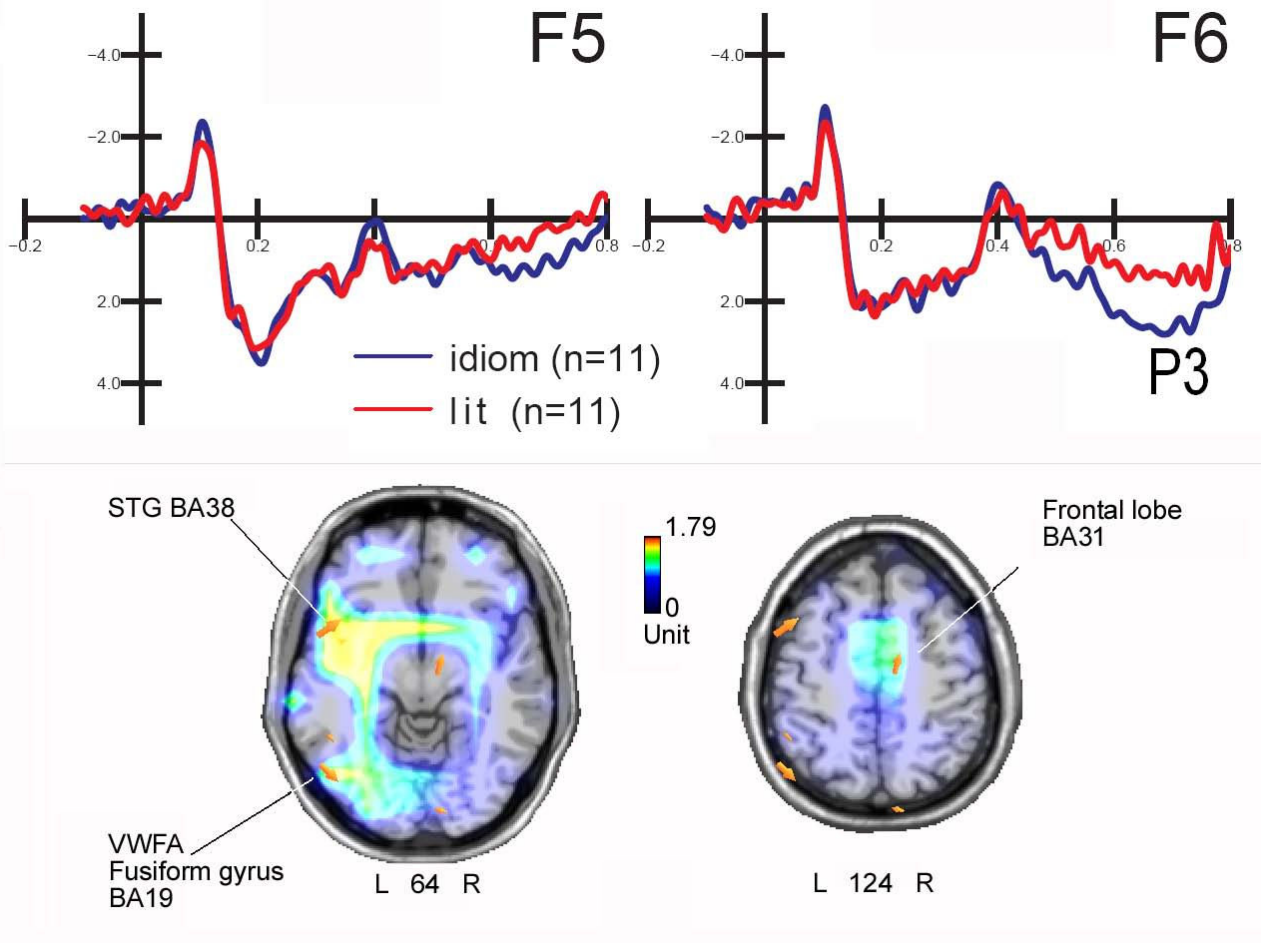
**Final words**. (Top) Grand-averaged ERPs recorded from left and right inferior frontal sites in response to idiomatic and literal phrases. This indicates the strong right-hemispheric lateralization of the anterior P3 component to idiomatic phrases. (Bottom) Two axial sections (deepness indicated below the head) of swLORETA inverse solution performed on the difference wave obtained by subtracting ERPs to literal from ERPs to idiomatic sentences in the time window 620-640 ms, corresponding to the peak of anterior late positivity. Grid spacing = 10 mm; estimated SNR = 3. The solution offered three strong sources of statistically significant activation explaining the surface difference-potential, one located in the left fusiform gyrus (BA 19), one in the left superior temporal gyrus (BA38), and one in the right medial frontal gyrus (BA9).

This component showed a hemispheric asymmetry (F1,10 = 11.93; p < 0.006), with much larger amplitudes over the right (3.52 μV) than the left (2.86 μV) hemisphere, as clearly appreciable in Figure 4 (top). The amplitude of this component was strongly affected by "sentence type", both per se (F1,10 = 9.92; p < 0.01) and in interaction with "electrode" (F1,10 = 5.74; p < 0.04) and "hemisphere" (F1,10 = 6.94; p < 0.025). Post-hoc comparisons indicated more positive responses to idiomatic than literal phrases only over the right hemisphere at inferior frontal sites, and bilaterally at mesial frontal sites.

In order to identify the possible intracranial generator of this effect, a swLORETA source reconstruction was performed (Figure 4, bottom) on the difference wave obtained by subtracting ERPs to literal from ERPs to idiomatic phrases in the time window 620-640 ms, corresponding to the response peak. This contrast allowed us to observe which brain regions were involved in the extra response to idiomatic expressions, and possibly to identify the right-hemispheric location of some neural generator(s). Table 5 provides a list of significantly active sources that could explain the different surface voltages and the Tailarach coordinates of their corresponding neural generators. Indeed, the inverse solution indicated, among other things, an active source in the right medial frontal gyrus (X = 11, Y = -14, Z = 45) for processing figurative language.

**Table 5:** Final words.

**Magn**.	**T-x**	**T-y**	**T-z**	**Hem**.	**lobe**	**area**	**BA**
10.64	-48	-66	-11	LH	Temporal	Fusiform gyrus	19

10.79	-48	8	-20	LH	Temporal	Superior temporal gyrus	38

10.35	11	-14	45	RH	Frontal	Medial frontal gyrus	9

7.28	-48	-51	42	LH	Parietal	Inferior parietal lobule	40

List of intracranial generators (along with their Tailarach coordinates) explaining the surface voltage Idiomatic-Literal final words in the 620-640 ms time window (slow wave component), according to swLORETA (ASA); grid spacing = 10 mm.

#### ERPs to associated/non-associated target words

**Figure 5:**
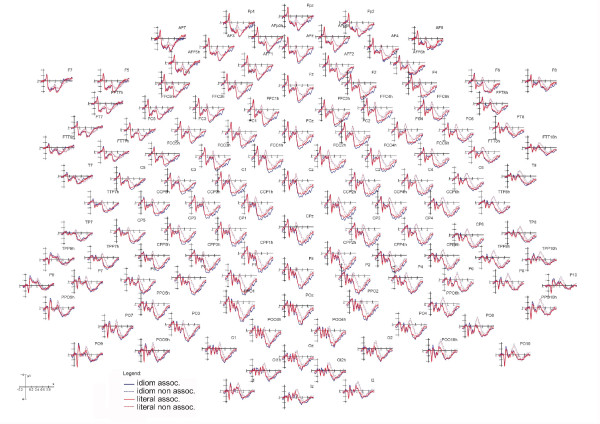
**Target words**. Grand-averaged ERPs recorded from all scalp sites as a function of sentence type and final word associativeness.

Figure 5 shows the grand-average ERPs recorded in response to idiomatic vs. literal expressions over all 128 scalp sites. Inspection of the ERP waveforms indicated that "sentence type" and "semantic associativeness" had strong effects after 350 ms of latency post-stimulus, a time range corresponding to the anterior and posterior P3 and N400 components.

#### Posterior N400 (380-480 ms)

**Figure 6:**
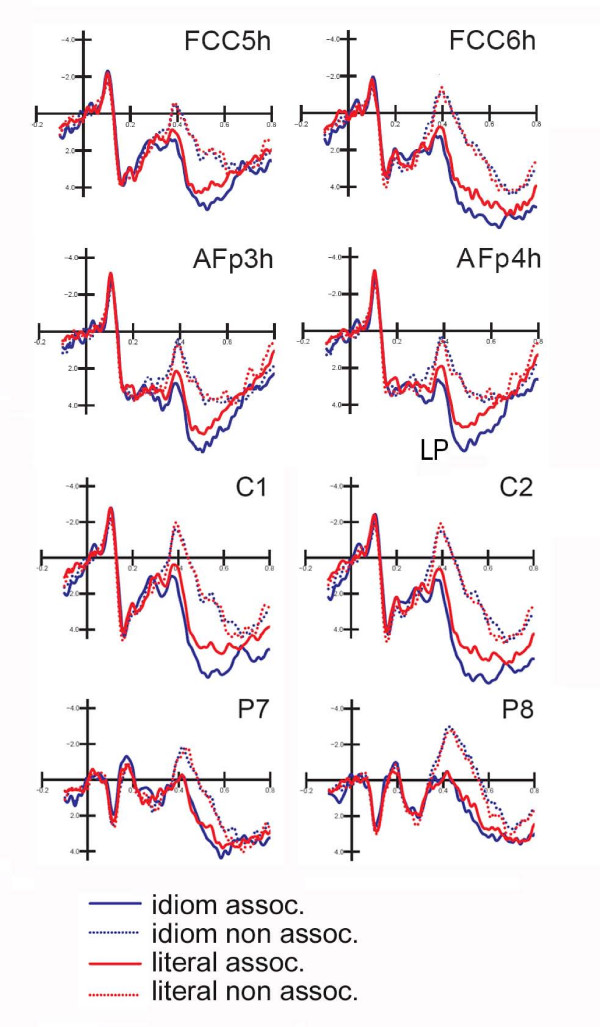
**Target words**. Grand-averaged ERPs recorded from occipito/temporal, mesial central, fronto-central and anterior-frontal sites as a function of sentence type and final word associativeness.

This negative posterior component was strongly modulated by "associativeness" (F1,10 = 34.28; p < 0.00016) with much larger negative responses to non-associated (-1.65 μV) than associated (1.5 μV) words. N400 was larger at parietal (P7, P8) than central (C1, C2) sites, as demonstrated by the significance of "electrode" (F1,10 = 5.8; p < 0.036). The significant interaction of "associativeness" and "hemisphere" (F1,10 = 6.36; p < 0.03) and relative Tukey post-hoc comparisons indicated bilateral positivity to associated words and a greater N400 over right than left centroparietal sites for non-associated words. There was no effect of sentence type on N400 amplitudes, as visible in Figure 6.

#### Anterior N400 (410-510 ms)

The anterior negative deflection showed the significance of "associativeness" (F1,10 = 48.23; p < 0.00005) with greater N4 responses to non-associated (0.59 μV) than associated (3.38 μV) target words. The interaction with the "electrode" factor (F1,10 = 63.71; p < 0.000001) indicated greater associativeness effects at frontal than anterior frontal sites (also visible in Figure 6).

#### Late positivity (500-650 ms)

The analysis of late positivity showed that "associativeness" was significant (F1,10 = 45.97; p < 0.00005), with larger LP to associated (4.7 μV) than non-associated (2.5 μV) targets; the further interaction with "electrode" (F1,10 = 29; p < 0.0003) indicated larger "associativeness" effects at frontal sites. The interaction of "associativeness" with "hemisphere" (F1,10 = 5.3; p < 0.05) and relative post-hoc comparison indicated more consistent effects over the right (ASS = 5.15 μV, Non-A.= 2.5 μV) than the left (ASS = 4.2 μV, Non-A.= 2.5 μV) hemisphere. The further interaction sentence type × associativeness × hemisphere (F1,10 = 5,27; p < 0.0045) indicated more positive responses to target words associated with idiomatic (5.41 μV) than literal (4.93 μV) expressions over the right hemisphere (see waveforms in Figure 6).

## Discussion

In this study, RTs to target words were longer when related to a idiomatic meaning than to a literal meaning. Since the target words were carefully matched across categories for length, frequency of use and abstractness, and the RT advantage was found for both associated and non-associated targets, a difference in task difficulty for idiomatic phrases might be hypothesized. A similar pattern of results has been found in a behavioural study [27] in which the participants were presented with incomplete priming sentences followed by literal or metaphorical lateralized target words and were asked to decide whether each sentence was literally true or false. The results showed that responses to metaphorical sentences were slower for stimuli in either the left (RH) or the right (LH) visual field. On the other hand, an electrophysiological study [20] involving a semantic decision task on meaningful or unrelated literal or metaphorical word pairs showed no difference in RTs (related literal word pairs = 873 ms; metaphorical pairs = 880 ms) between the two classes, suggesting that task requirements have a specific role in inducing possible costs for metaphorical language. Again, in an ER-fMRI study [16] in which the participants were asked to evaluate whether metaphorical or literal sentences were meaningful or meaningless (e.g. metaphorical (*Some surgeons are butchers*), literal (*Some surgeons are fathers*) or non-meaningful (*Some surgeons are shelves*)) showed no significant difference between processing times for literal and idiomatic sentences.

Other studies [28] have even found faster RTs to figurative than literal targets in strongly salient conditions, or to predictable idiomatic than literal ending terms [29]. On the other hand, the ERP study by Coulson and Van Petten [14] showed a larger amplitude N400 and lower accuracy for low-cloze metaphorical than literal final parts.

In our study, the ERP data recorded in response to associated target words indicated a larger anterior positivity to words semantically related to idiomatic than to literal phrases. These results can be interpreted as evidence for a more effortful demand in semantic-association tasks based on idiomatic meaning-codes. The increased workload might be determined by the need to switch from the figurative semantic domain of idiomatic keys to the strictly literal semantic domain of the defining target words, especially if one assumes a difference in neurofunctional circuits subserving the two types of knowledge. In addition, a methodological factor cannot be excluded, which, while intrinsic to the nature of the stimulus (and therefore hardly avoidable), was still not controlled for; that is, the different degrees of plain semantic relatedness between target words and their contexts. For example, the semantic relatedness between "gloves" and "kindness" is obviously different from that between "antibiotics" and "therapy" (for the two sentences "I have been treated with (kid) gloves" and "I have been treated with antibiotics"). This might in principle entail greater difficulty for semantic decisions based on figurative material, thus resulting in slower RTs (especially for associated words) and eliciting stronger prefrontal LP responses reflecting the more demanding task condition (uniquely for associated items). The lack of prefrontal involvement and sentence type effect for LP responses recorded in response to non-associated words further support the above hypothesis. Clearly, this potential problem had no effect on the ERP signals recorded in response to final phrases, since they just reflected time-locked comprehension processes. Besides, each linguistic or semantic variable was controlled for across stimulus categories.

As for electrophysiological measures, the first component displaying an effect of sentence type was the occipito/temporal N2, the amplitude of which was greater in response to idiomatic than literal expressions over the left hemisphere. Its neural generators (according to swLORETA) included the left and right fusiform areas (BA19/37), with a strong left hemispheric asymmetry. The present data support the view that there is direct access to the idiomatic meaning of figurative language, not dependent on the suppression of its literal meaning. Accordingly, they do not support the "indirect access hypothesis" [18]. Indeed, early linguistic processing areas, namely the left fusiform gyrus (BA19) possibly representing the Visual Word Form Area [30], showed significant differences in the activity elicited by literal vs. idiomatic final words at early latency stages. These early effects of semantic stimulus content on the activity of the left fusiform gyrus at this processing stage are not surprising if one consider that other studies have shown that the same area is sensitive to words lexical properties at even earlier latency levels [31], with larger N2 responses to high than low frequency words or to words than pseudowords [32]. However, since idiomatic and literal final words were matched for orthographic and lexical properties (length, familiarity cloze probability, frequency of use, abstractness), N2 effect (which according to LORETA indexed the activity of bilateral FG and right medial frontal gyrus) probably reflected the metaphorical (or not prosaic) nature of meanings accessed.

The final words of idioms elicited larger N400 than those of the literal control sentences, as in other ERP studies [33]. The larger N400 to idiomatic than to literal final words is consistent with the more recent ERP findings by Coulson and Van Petten [14], who found a larger N400 to low-cloze metaphorical than literal expressions. The authors interpreted those data in terms of the greater difficulty of metaphorical interpretation. Indeed, their study consisted in performing a semantic comprehension task by responding to a simple question after sentence reading. In the present study, the larger N400 for idiomatic versus literal expressions is most likely due to their figurative or literal nature, rather than any difference in cloze probability since it was matched across stimuli. As for the neural generators of N400 surface activity, according to the swLORETA inverse solution, the contrast idiomatic-minus-literal revealed a difference in the activation of a series of regions including the left and right occipital and temporal cortex of the fusiform gyri, the right middle temporal gyrus (BA21), the right parahippocampal gyrus, the left cingulate gyrus and the left middle frontal gyrus (BA49). It might be hypothesized that the greater activation of limbic areas is related to the greater emotional connotation of figurative language. Indeed some authors have found a greater activation of cingulate cortex and the hippocampal formation during processing of affective words [34-36]

The role of the right superior temporal area in the processing of idiomatic expressions seems to be a finding common to many studies [15,18,20-23]. For example, Bottini and colleagues [21] performed a PET study comparing metaphorical and literal comprehension of sentences. Comprehension of metaphors was associated with similar activations in the left hemisphere, but in addition, a number of sites were activated in the right hemisphere: the prefrontal cortex, the middle temporal gyrus, the precuneus and the posterior cingulate. Activation of the right middle temporal gyrus for processing idiomatic language was also found by fMRI scanning [15] and in the combined ERP/LORETA study by Arzouan and colleagues [20]. In the latter study, Hebrew readers were required to decide whether each item in a series of literal or metaphorical semantically-related or -unrelated word pairs conveyed a meaning. The contrast between metaphorically-related and literally-related word pairs revealed the activation of a cluster of voxels including the right middle and superior temporal gyri, the right fusiform gyrus and the right inferior parietal lobule, plus a smaller activation of the right inferior frontal gyrus. Similarly, the right superior temporal gyrus and right middle frontal gyrus, among other areas, were also found to be activated for processing of metaphorical language in recent fMRI studies [18,22]. However, this corpus of data is not consistent with an rTMS study [17], which showed that if stimulation of the left inferior frontal gyrus disrupts the processing of familiar metaphors, stimulation of the posterior part of the right STS does not impair the processing of conventional but only of novel metaphorical word pairs.

In our study, the involvement of the medial prefrontal cortex during comprehension of idiomatic sentences was considerable, which is consistent with the notion that the processing of idiomatic language is a prefrontal task and especially a right prefrontal one [16,22]. However, we found no specific activation of the left inferior frontal (IF) gyrus, as in several other studies [15,18,20], whereas according to our data the prefrontal activation (BA9) was strictly right-sided in the N2 and LP latency ranges. We believe that the lack of left IF activation might be because ERPs time-locked to sentence final words were not response-related (at that moment, the task consisted in comprehending the phrase and integrating it with the previous semantic context). The left inferior frontal gyrus seems instead to be more involved in tasks requiring selection among competing representations (as also in verb generation, response inhibition, response selection tasks [37,38]) as required in picture/sentence matching tests (e.g [18]. In fact, while there is no doubt that the left inferior frontal gyrus (LIFG) is involved in sentence-level syntactic processing, as also supported by Broca-related deficits in grammatical processing for either production or comprehension [39], it has been proposed [38] that LIFG contribution to sentence-level syntactic processing might be more specifically related to its role in the detection and resolution of incompatible representations, which enable the reanalyses of syntactically complex sentences, and generally speaking, overriding highly regularized, automatic processes. Therefore, the lack of left IFG activation in our study supports the notion that the comprehension of idiomatic language does not require the inhibition or suppression of the corresponding literal representation. At this regard, it can be interesting to consider that, while some studies advanced the hypothesis that metaphor comprehension is based on the suppression of metaphor-irrelevant information [40], other studies have provided evidence that this is not the case. Specifically, Monetta and Pell [41] investigated metaphor comprehension processes in Parkinson patients who typically show an impairment in the pragmatic functions of language such as metaphor comprehension, and found no evidence of a specific impairment in the ability to suppress metaphor irrelevant information as for example the fact that "*Roses have thorns" *relative to the sentence "*That baby's cheeks are roses*". Instead they argued for a crucial role of the fronto-striatal system in "complex" forms of language processing such as metaphor interpretation, strongly involving working memory functions.

Indeed, notwithstanding that older adults may have difficulty inhibiting irrelevant or contextually inappropriate information in text comprehension and recall, they show no difference with younger controls in priming tasks comparing metaphor-relevant vs. literal-relevant sentence processing [42]. This finding show either that inhibiting processing in elderly are specifically preserved as far as metaphor comprehension, either that metaphor comprehension does not imply the suppression of literal meaning, as our data seem to confirm.

## Conclusion

In summary, high temporal resolution ERP data (compared to rTMS and fMRI) suggest that both hemispheres are involved in the processing of idioms. Several left and right structures were simultaneously active at different processing stages, in a temporal sequence that mainly engaged the left hemisphere during an early phase (e.g. the left fusiform gyrus and the left and right medial frontal gyri as early as 250 ms post-stimulus) and subsequently more bilateral areas, with larger effects over the right hemisphere at anterior regions (such as the right middle temporal gyrus (BA21) at about 400 ms and the right medial frontal gyrus from about 500 ms on). The lack of activation of the left inferior frontal area, interpreted by some authors as responsible for the suppression of the sentence literal meaning, in our study supports the view that the comprehension of idiomatic sentences does not require the inhibition of the corresponding literal representation. Overall, on the basis of our data, we conclude that the interpretation of language involves widespread distributed systems bilaterally, with the right hemisphere having a special role in the evaluation/comprehension of idiomatic meaning. In addition, the specific activation of left and right limbic regions, including the cingulate and parahippocampal cortex for the idiomatic-literal contrast (400-450 ms), suggest that they have a role in providing the emotional connotation of colourful idiomatic language.

## Authors' contributions

AMP conceived and designed the study, interpreted the data and wrote the manuscript. NC contributed to study's design and planning, prepared the stimuli and performed ERP analyses. RA was involved in EEG recording and source reconstruction. AZ participated in the data evaluation and interpretation. All authors read and approved the final manuscript.
